# Suicide Among Physicians and Medical Students in Bangladesh: Variations in Associated Factors

**DOI:** 10.1002/puh2.70243

**Published:** 2026-04-21

**Authors:** S. M. Yasir Arafat, Sadeed Hossain, Farhin Islam

**Affiliations:** ^1^ Department of Psychiatry Bangladesh Specialized Hospital Dhaka Bangladesh; ^2^ Department of Public and Community Health, Faculty of Medicine and Health Sciences Frontier University Garowe Garowe Somalia; ^3^ Biomedical Research Foundation Dhaka Bangladesh; ^4^ Bangladesh Institute of Development Studies (BIDS) Dhaka Bangladesh

**Keywords:** academic pressure, doctor suicide, medical student suicide, physician suicide, suicide in Bangladesh

## Abstract

**Background:**

Although health professionals and medical students may be at higher risk for suicide due to occupational stress, academic pressure, and access to lethal methods, evidence regarding suicide among these groups in Bangladesh remains limited.

**Objective:**

To examine and compare suicide methods, locations, and reported associated factors between medical students and physicians in Bangladesh.

**Methods:**

This media‐based retrospective descriptive study identified 50 suicide cases (25 medical students and 25 physicians) from Bangladeshi online news reports published between 2010 and 2025. Data on sociodemographics, suicide methods, locations, timing, and reported associated factors were extracted. Categorical variables were analyzed using chi‐square or Fisher's exact test, and continuous variables using an independent samples *t*‐test. A *p* value <0.05 was considered statistically significant.

**Results:**

Hanging was the most common method (57.8% among cases with available method information, *n* = 45), followed by poisoning/overdose (33.3%), with no significant differences between the groups. Associated risk factors showed marked differences: Academic pressure and exam failure were predominant among students (71.4%), whereas marital discord was most common among physicians (42.1%, *p* < 0.001).

**Conclusions:**

The findings suggest differences in reported associated factors between medical students and physicians in Bangladesh. Academic stress appeared more frequently among students, whereas marital discord was more commonly reported among physicians. These preliminary findings may help inform future suicide prevention initiatives targeting different career stages within the medical profession.

## Graphical abstract

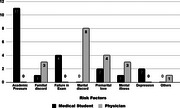

## 1 Introduction

Suicide is a major global public health concern. According to the World Health Organization (WHO), more than 720,000 people take their own lives each year by various methods. Although people of any age range could die by suicide, in 2021 globally it was the third leading cause of death among 15–29‐year‐olds [1]. Health professionals and medical students are the high‐risk populations due to their exposure to occupational stress, emotional exhaustion, academic pressure, knowledge of lethal methods, and easy access to potentially lethal means [2]. They had higher suicide rates compared to the general population. One study reported suicide as the second most common cause of death for medical students [3], whereas another study stated that physicians, particularly female physicians, had higher suicide rates than the general population [4].

The method of suicide is important because it is directly related to the lethality and prevention feasibility of suicide [5]. The choice of methods of could be influenced by various factors such as their profession, gender, availability of lethal tools, and social or cultural beliefs about certain methods [6, 7]. The rate of suicide could be reduced by limiting access to common or highly lethal means. Suicide rate could be reduced by means restriction, even if no other interventions are introduced [8].

Studies from high‐income countries showed that physicians commonly die by suicide by self‐poisoning or overdose [9], whereas medical students more commonly died by hanging [10]. Similar findings were found in Bangladesh [11, 12]; however, comparison between occupational groups and educational variations were rarely studied in the country, leaving an important gap in understanding suicide patterns.

In Bangladesh, suicide is considered a criminal act, there is no national suicide data of Bangladesh [13]. Therefore, proper information about the distribution of age, sex, occupation, religion of deceased, and methods of suicide is lacking, which is a fundamental challenge for suicide prevention [14]. A recent study had examined suicide methods in specific professional groups (police officers and physicians), which identified variations in the methods of suicide between the police and physicians [11]. Another case series reported suicides and associated factors among medical students [12]. A recent study assessed suicide among medical students and doctors (*n* = 37), collecting data from media reports for suicides between 2020 and 2024 [15]. However, no comparative investigation has been conducted to assess the differences in suicide methods between the groups. These two groups are linked by the same professional culture but are different in age and risk factors. Understanding these variations could help to develop targeted prevention strategies. It could increase focusing on proper means restriction, early identification of distress, and institutional support systems. Therefore, this study aimed to compare the methods, places, and risk factors of suicide among medical students and physicians in Bangladesh.

## 2 Methods

### 2.1 Study Design

This media‐based retrospective descriptive study examined suicide patterns among medical students and physicians in Bangladesh. The study focused on identifying and analyzing reported suicides from online media sources in Bangladesh.

### 2.2 Data Collection

Data were extracted from Bangladeshi online news reports between 12 and 14 November 2025 with specific search terms to identify the suicides among medical students. The search was conducted on Google by using both Bangla and English terms such as “Suicide in doctors,” “suicide in physicians,” “suicide in medical students.” We used the dataset was obtained from a previously conducted study [11], which used similar sources and inclusion criteria for the list of suicides of physicians.

The Google search retrieved the news reports all the available news portals and online version of the newspapers. We identified reports from widely used Bangladeshi online news portals, including Prothom Alo, The Daily Star, Dhaka Tribune, BDNews24, Jugantor, and Somoy News. The search covered suicide events reported between 2010 and 2025.

### 2.3 Inclusion and Exclusion Criteria

Suicides among Bangladeshi citizens living in Bangladesh were included. Moreover, cases that clearly identified the individual as a medical student or physician were included.

Suicides of foreigners and suicides of Bangladeshi citizens living abroad were excluded. As data were extracted from online news reports, multiple reports of a suicide were published in multiple outlets. When multiple news reports described the same suicide case, duplicates were identified and removed based on name, age, date, and location of the incident.

### 2.4 Data Extraction

For each eligible case, information of the date of suicide, name, age, sex, religion, method of suicide, location of the attempt, time of the attempt, and any reported associated factors was extracted by using Microsoft Excel 2019.

Reported associated factors refer to circumstances mentioned in the media reports that were described as contributing to the suicide event, such as academic pressure, examination failure, marital discord, familial conflict, romantic relationship problems, or mental health conditions. These factors were recorded only when explicitly stated in the news reports and therefore represent reported circumstances rather than clinically verified risk factors.

### 2.5 Statistical Analysis

Statistical analysis was performed using Stata (version 14). Frequencies and percentages were used to summarize the categorical variables of the study sample, and continuous variables were mentioned as means and standard deviations. The bivariate associations between profession (medical student and physician) and categorical variables were measured by performing the chi‐square test. Fisher's exact test was conducted for those categorical variables that had an expected frequency of less than five observations in a cell. Independent samples *t*‐test were used to ascertain the bivariate associations between profession and a continuous variable (age in years). Two‐sided test was done and a *p* value of <0.05 was considered statistically significant.

### 2.6 Ethical Considerations

As the study used publicly available media reports, therefore, no formal ethical approval for this study was taken.

## 3 Result

### 3.1 Sociodemographic Characteristics

As shown in Table 1, a total of 50 suicide cases were identified, where there were 25 medical students and 25 physicians. As expected, medical students were younger and predominantly unmarried, whereas physicians were older and more frequently married. No significant differences were observed in sex or religion between the groups.

**TABLE 1:** Characteristics suicide among medical students and physicians in Bangladesh.

Variable	Total *n* (%)	Medical students *n* (%)	Physicians *n* (%)	*χ* ^2^	*p* value
**Sex**	*n* = 50	*n* = 25	*n* = 25		
Male	26 (52.00)	13 (52.00)	13 (52.00)	0.0000	1.000^a^
Female	24 (48.00)	12 (48.00)	12 (48.00)		
**Age (years)**	*n* = 36	*n* = 16	*n* = 20		
18–25	15 (41.67)	14 (87.50)	1 (5.00)	24.8914	<0.001^a^
>25	21 (58.33)	2 (12.50)	19 (95.00)		
Mean (±SD) age (years)	29.4 (9.78)	22.9 (1.86)	34.6 (10.44)	4.4243	<0.001^b^
**Marital status**	*n* = 49	*n* = 25	*n* = 24		
Ever married	17 (34.69)	2 (8.00)	15 (62.50)	16.0525	<0.001^a^
Unmarried	32 (65.31)	23 (92.00)	9 (37.50)		
**Religion**	*n* = 50	*n* = 25	*n* = 25		
Muslim	36 (72.00)	18 (72.00)	18 (72.0)		0.41^c^
Hindu	12 (24.00)	5 (20.00)	7 (28.0)		
Buddhist	2 (4.00)	2 (8.0)	0 (0.0)		
**Method**	*n* = 45	*n* = 21	*n* = 24		
Hanging	26 (57.78)	14 (66.67)	12 (50.0)		0.73^c^
Poisoning/Overdose	15 (33.33)	6 (28.57)	9 (37.50)		
Jump from a height	2 (4.44)	1 (4.76)	1 (4.17)		
Burning	1 (2.22)	0 (0.00)	1 (4.17)		
Cut	1 (2.22)	0 (0.0)	1 (4.17)		
**Place of suicide attempt**	*n* = 45	*n* = 24	*n* = 21		
Home	33 (73.33)	15 (62.50)	18 (85.71)		0.040^c^
Friends’ home	1 (2.22)	0 (0.00)	1 (4.76)		
Hostel	11 (24.44)	9 (37.50)	2 (9.52)		
**Time**	*n* = 41	*n* = 21	*n* = 20		
Morning	8 (19.51)	3 (14.29)	5 (25.00)		0.081^c^
Noon	5 (12.20)	5 (23.81)	0 (0.00)		
Evening	7 (17.07)	2 (9.52)	5 (25.00)		
Night	21 (51.22)	11 (52.38)	10 (50.00)		
**Risk factors**	*n* = 40	*n* = 21	*n* = 19		
Academic pressure	11 (27.50)	11 (52.38)	0 (0.00)		<0.001^c^
Familial discord	4 (10.00)	1 (4.76)	3 (15.79)		
Failure in exam	4 (10.00)	4 (19.05)	0 (0.00)		
Marital discord	8 (20.00)	0 (0.00)	8 (42.11)		
Premarital love	6 (15.00)	2 (9.52)	4 (21.05)		
Mental illness	4 (10.00)	1 (4.76)	3 (15.79)		
Depression	2 (5.00)	2 (9.52)	0 (0.00)		
Others	1 (2.50)	0 (0.00)	1 (5.26)		
Suicide note	*n* = 50	*n* = 25	*n* = 25		
Present	10 (20.00)	4 (16.00)	6 (24.00)	0.5000	0.48^a^
Absent	40 (80.00)	21 (84.00)	19 (76.00)		

Abbreviation: SD, standard deviation.

^a^Pearson's chi‐square.

^b^Independent samples *t*‐test.

^c^Fisher's exact test.

The location of suicide attempts had significant differences (*p* = 0.04). Most of the physicians died by suicide at their own home (85.7%), whereas only 62.5% medical students attempted it in their home. Hostel‐based suicides were more common among medical students (37.5%) than physicians (9.5%). However, the temporal pattern of suicide attempts showed no significant differences. Night time was the most common period for suicide in both groups (51.2%; *p* = 0.08).

### 3.2 Methods of Suicide

Information on the method of suicide was available for 45 of the 50 identified cases. Among these cases, hanging was the most common method (57.8%), followed by poisoning or sedative overdose (33.3%). There were no statistically significant differences in suicide methods between medical students and physicians. Burning and cutting were observed only among physicians.

### 3.3 Associated Factors of Suicide

**FIGURE 1:**
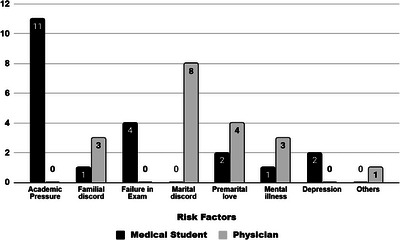
Contributing factors for suicide among medical students and physicians in Bangladesh.

The statistically significant difference was noted in the reported associated factors of suicide between two groups (*p* < 0.001). Academic pressure was the leading factor among medical students, around 52.4% of medical students died by suicide due to academic causes. However, none of the physicians did it due to academic pressure. Similarly, failure in examinations was reported in 19.1% of medical student suicides but absent among physicians. In contrast, marital discord was the most common reported associated factor among physicians (42.1%) and was not reported for any medical student. Other factors such as familial discord, premarital relationship issues, and mental illness appeared in both groups but with low frequencies. Findings are shown in Figure 1 and Table 1.

### 3.4 Suicide Notes

Suicide notes were found in 20% of all cases, with no significant difference between medical students and physicians (*p* = 0.48).

## 4 Discussion

### 4.1 Major Findings

This is one of the first comparative assessments of suicide characteristics among medical students and physicians in Bangladesh. Although both groups belong to the same professional spectrum, significant differences were observed in age, marital status, risk factors, and locations of suicide attempts.

In this study, we found significant differences in life events of suicide between medical students and physicians. Academic‐related stressors, including academic pressure and exam failure, were more common (71.43%) among medical students, whereas marital and familial discord emerged as the predominant factor (57.90%) among physicians. This finding suggests that students may be more vulnerable to academic failure and institutional pressure, whereas physicians face increased relational and family stress as adults with professional and marital responsibilities. We also found variation in other associated factors, such as familial discord, premarital love, and mental health conditions, although these occurred less often. The overall pattern suggested that while emotional and interpersonal factors were prominent across both groups, the primary risk factors were academic stress for medical students and marital conflict for physicians. These results align with previous investigations among Indian and Bangladeshi medical students and physicians, where academic challenges and exam‐related distress were major precipitants of suicide for medical students and marital discord was for physicians [15, 16].

Hanging was the predominant method in both groups, followed by poisoning or sedative overdose. This aligned with national patterns in Bangladesh, where hanging and poisoning were widespread due to their accessibility, cultural familiarity, and high lethality [17]. Another previous study among the same population found similar pattern [15].

Medical students were significantly younger than physicians, with most aged 18–25 years, whereas the majority of physicians were older than 25 years. This pattern aligned with global studies showing that younger medical trainees experience high academic pressure and academic stress was found to be risk factors for suicidal ideation of medical students [18, 19]. Physicians were more frequently married (62.50%), and around 42% died by suicide due to marital discord. Similar findings were found in other global studies [16, 20].

### 4.2 Implications of Study Results

These findings indicate several important implications. First, the study highlighted the need to address associated factors specific to each career stage. For medical students, the strong influence of academic stress underscored the importance of mentoring and counseling following academic failure. Second, for physicians, the predominance of marital and familial discord highlighted the need for relationship counseling services, workplace‐based psychosocial support, and programs that promote work–life balance. Overall, these findings emphasized that suicide prevention could be context‐sensitive, considering both professional and personal risk factors for both groups.

### 4.3 Strengths and Limitations

This study is the first to compare suicide characteristics between medical students and physicians in Bangladesh using media reports, contributing valuable data in a context where national suicide statistics are lacking.

However, there are limitations that need to be acknowledged. First, media reports may contain incomplete or inaccurate information, and not all suicide cases are reported in the media. Therefore, the dataset may be affected by selection bias and reporting bias, which limits generalizability of the findings. Second, the sample size was small, which may limit generalization of the study findings. Third, some sociodemographic variables were not reported properly which limited the ability to explore all potential associations. Despite these limitations, the study provided novel insights into occupational and educational variations in suicide patterns of Bangladesh, which would help to develop targeted prevention strategies in Bangladesh.

## 5 Conclusion

This study suggests potential differences in suicide patterns between medical students and physicians in Bangladesh. Academic stress and failure in exam appeared more frequently among medical students, whereas marital and familial discord were predominant among physicians. These findings suggest the need for career‐stage–specific prevention measures. For medical students, interventions should focus on reducing academic pressure through mentoring, counseling, and institutional support. For physicians, promoting work–life balance, relationship counseling, and mental health support may mitigate suicide risk. Overall, the study emphasizes that both group‐specific and universal psychosocial factors must be considered to develop effective suicide prevention programs in Bangladesh.

## Author Contributions

Conception: S. M. Yasir Arafat. Data curation: S. M. Yasir Arafat and Sadeed Hossain. Data analysis: Farhin Islam. Writing – original draft: S. M. Yasir Arafat and Sadeed Hossain. Writing – review and editing: S. M. Yasir Arafat, Sadeed Hossain, Farhin Islam. All authors have read and approved the final version of the manuscript.

## Funding

The authors have nothing to report.

## Conflicts of Interest

The authors declare no conflicts of interest.

## Data Availability

Data available on request from the authors.
